# Prediction of mild cognitive impairment using blood multi-omics data

**DOI:** 10.3389/fgene.2025.1552063

**Published:** 2025-05-26

**Authors:** Daniel Frank Zhang, Cigdem Sevim Bayrak, Qi Zeng, Minghui Wang, Bin Zhang

**Affiliations:** ^1^ Mount Sinai Center for Transformative Disease Modeling, Icahn School of Medicine at Mount Sinai, New York, NY, United States; ^2^ Department of Computer Science, Rice University, Houston, TX, United States; ^3^ Department of Genetics and Genomic Sciences, Icahn School of Medicine at Mount Sinai, New York, NY, United States; ^4^ Department of Pharmacological Sciences, Icahn School of Medicine at Mount Sinai, New York, NY, United States; ^5^ Department of Artificial Intelligence and Human Health, Icahn School of Medicine at Mount Sinai, New York, NY, United States; ^6^ Icahn Institute of Genomics, Icahn School of Medicine at Mount Sinai, New York, NY, United States

**Keywords:** mild cognitive impairment, Alzheimer’s disease, machine learning, copy number variation, gene expression

## Abstract

Mild cognitive impairment (MCI) represents an initial phase of memory or other cognitive function decline and is viewed as an intermediary stage between normal aging and Alzheimer’s disease (AD), the most prevalent type of dementia. Individuals with MCI face a heightened risk of progressing to AD, and early detection of MCI can facilitate the prevention of such progression through timely interventions. Nonetheless, diagnosing MCI is challenging because its symptoms can be subtle and are easily missed. Using genomic data from blood samples has been proposed as a non-invasive and cost-efficient approach to build machine learning predictive models for assisting MCI diagnosis. However, these models often exhibit poor performance. In this study, we developed an XGBoost-based machine learning model with AUC (the Area Under the receiver operating characteristic Curve) of 0.9398 utilizing gene expression and copy number variation (CNV) data from patient blood samples. We demonstrated, for the first time, that data at a genome structure level such as CNVs could be as informative as gene expression data to classify MCI patients from normal controls. We identified 149 genomic features that are important for MCI prediction. Notably, these features are enriched in the pathways associated with neurodegenerative diseases, such as neuron development and G protein-coupled receptor activity. Overall, our study not only demonstrates the effectiveness of utilizing blood sample-based multi-omics for predicting MCI, but also provides insights into crucial molecular characteristics of MCI.

## Introduction

Alzheimer’s disease (AD) is the most common type of dementia and is characterized by memory impairment and other cognitive problems. Its pathological signature is marked by the presence of amyloid beta (Aβ) plaques, neurofibrillary tangles (NFT), neuronal death, and synaptic loss (Long and Holtzman, 2019). Approximately 6.7 million Americans aged 65 and older are living with AD. While the number of Americans with AD is projected to be 13.8 million by 2060, current treatments can only delay the progression of the disease, and no therapeutics can reverse the progression (Alzheimer’s Association Report, 2023).

AD typically progresses slowly, often worsening over a decade or more. Over time, enough brain damage accumulates to lead to cognitive symptoms and impairment. Mild cognitive impairment (MCI) is an early stage of the loss of memory or other cognitive ability (such as language or visual perception) and is considered to be a transitional stage between normal aging and AD (Petersen, 2016). Studies show that MCI patients are at an increased risk of developing AD, at a yearly rate of 10%–15% (Petersen et al., 2009). Therefore, diagnosing MCI reliably can help to identify people who are likely to progress to AD. Patients diagnosed with MCI may receive treatments, advice, and support, aimed at reducing their risk of developing AD. For example, Lecanemab, an FDA-approved drug for the treatment of MCI due to AD, can moderately prevent the decline of cognition and function in MCI patients (van Dyck et al., 2023). Additionally, MCI patients can be monitored regularly, and dementia can be diagnosed earlier if they progress, allowing for more prompt treatment.

Diagnosing MCI is challenging because its symptoms are often subtle and its molecular mechanisms are not well-understood, making it difficult to differentiate from normal aging-related cognitive decline. Current diagnosis of MCI is based on tests with complex criteria, the core elements of which include thorough medical history; assessment of independent function and daily activities; input from a family member or trusted friend to provide additional perspective on how function may have changed; assessment of mental status using brief tests designed to evaluate memory, planning, judgment and other key thinking skills; evaluation of mood to detect depression and laboratory tests such as blood tests and imaging of the brain structure (Bradfield, 2023). It is important to understand that no single test can definitively diagnose MCI. Therefore, methods that can provide additional information about the patient’s memory or other cognitive functions could assist in clarifying the diagnosis.

Recently, machine learning has been used to predict MCI/AD with promising performance by searching for various features associated with the disease. However, most of these machine learning models are developed using data from patient brain tissues (e.g., gene expression), magnetic resonance imaging (MRI), and/or positron emission tomography (PET); methods that are either invasive or costly (Syaifullah et al., 2020; Sheng et al., 2022; Ahmed et al., 2022; Zhao et al., 2023). Studies have demonstrated that systemic changes in blood cells and plasma can reflect MCI/AD pathology in the brain (Humpel and Hochstrasser, 2011), suggesting that machine learning methods utilizing molecular profiles of patient blood samples could offer a non-invasive and cost-efficient alternative for predicting MCI, as well as understanding the potential molecular mechanisms of the disease. Indeed, researchers have reported some encouraging results. For example, AlMansoori et al. reported a Random Forest model trained on blood sample gene expression data predicting MCI/AD with AUC of 0.65 (AlMansoori et al., 2024). Oriol et al. utilized blood-derived SNPs and a BSWMS-LASSO-RPART ensemble classifier to predict AD patients with AUC of 0.72 (De Velasco Oriol et al., 2019). These prediction models frequently exhibit low accuracy, at least partly due to the noise intrinsic to the genome-wide molecular profiling data, therefore are not yet suitable for clinical application.

Studies have demonstrated that models trained on multi-omics data often achieve better performance than on single-omics data (AlMansoori et al., 2024; Gunther et al., 2012; Huang et al., 2019; Singh et al., 2019; Grueso and Viejo-Sobera, 2021). For instance, SNPs have been often used in combination with other types of data to improve prediction performance. Nonetheless, as mentioned earlier, the performance of these multi-omics methods is still lacking. As such, it could be useful to consider adding previously unconsidered omics data types into this multi-omics prediction paradigm.

Our previous study has identified MCI specific CNVs (Copy Number Variations), which measure genomic variations at a structural level instead of at a single nucleotide have change like SNPs (Ming et al., 2022). To our knowledge, CNVs not yet been utilized for AD/MCI prediction. Therefore, it is interesting to know how well MCI patients can be differentiated from those with normal cognitive function at a genome structure level and whether incorporating CNV data can improve machine learning performance for predicting MCI patients.

In this study, we developed a state-of-the-art model for the prediction of MCI patients using denoised whole genome gene expression and CNV data derived from the blood samples of patients with MCI and those without cognitive impairment in the Alzheimer’s Disease Neuroimaging Initiative (ADNI) cohort. Our study also shed light on MCI-associated molecular characteristics, especially genome structure level variations, which are essential for understanding and diagnosing the disease.

## Materials and methods

### Study participants

The data used in this study were obtained from the Alzheimer’s Disease Neuroimaging Initiative (ADNI) database. The ADNI launched in 2003 as a public-private partnership led by Principal Investigator Michael W. Weiner, MD. For each participant, blood data were recorded at their baseline visits, along with their MRI and DTI (Diffusion Tensor Imaging) data (Veitch et al., 2024). Each patient’s diagnosis during the baseline visits falls into one of the four disease statuses: no cognitive impairment (NCI), early mild cognitive impairment (EMCI), late mild cognitive impairment (LMCI), or Alzheimer’s disease (AD). Supplementary Table S1 shows the sample size of each disease status group in the gene expression and copy number variation data we used in this study. Since the focus of this study is the prediction of MCI, only the participants diagnosed as NCI or MCI (i.e., early MCI (EMCI) or late MCI (LMCI)) were included in this study.

Since we observed the significant age and sex difference between MCI and NCI groups, the data used in this study were adjusted for sex and age as described in the following data processing section.

### Data acquisitions and processing

#### Gene expression data

Gene expression data from the blood samples of 385 MCI patients and 221 normal healthy controls were generated by the ADNI study using Affymetrix Human Genome U219 platform (Affymetrix, Santa Clara, CA) (Saykin et al., 2015). For multiple probe sets corresponding to a particular gene, only the most varying one was selected to represent the gene while the probe sets that do not correspond to any gene or correspond to multiple genes were excluded. The processed data was then corrected for gender, age, ethnicity, cohort, RNA integrity number (RIN), and batch effects by linear regression. The gene expression data was preprocessed as previously reported (Wang et al., 2018).

#### Copy number variation (CNV) data

Autosomal CNVs were identified from the whole-genome sequencing (WGS) data from the ADNI study using four complementary CNV calling pipelines including CNVnator, Pindel, MetaSV, and Delly2, as previously described (Ming et al., 2022). Individual-level CNV calls from the four methods were integrated into a set of population-level CNVs. Consensus of CNVs detected by three or more methods were used for the subsequent analyses. A total of 24,166 autosomal CNVs including 16,723 deletions, 2,084 duplications, and 5,359 multi-allelic CNVs were identified. The data was adjusted for sex, age, ethnicity, and cohort. Bedtools and GRCh37 human genome were used to map the CNV genome locations to gene names.

### Preparation of training and testing data

**FIGURE 1:**
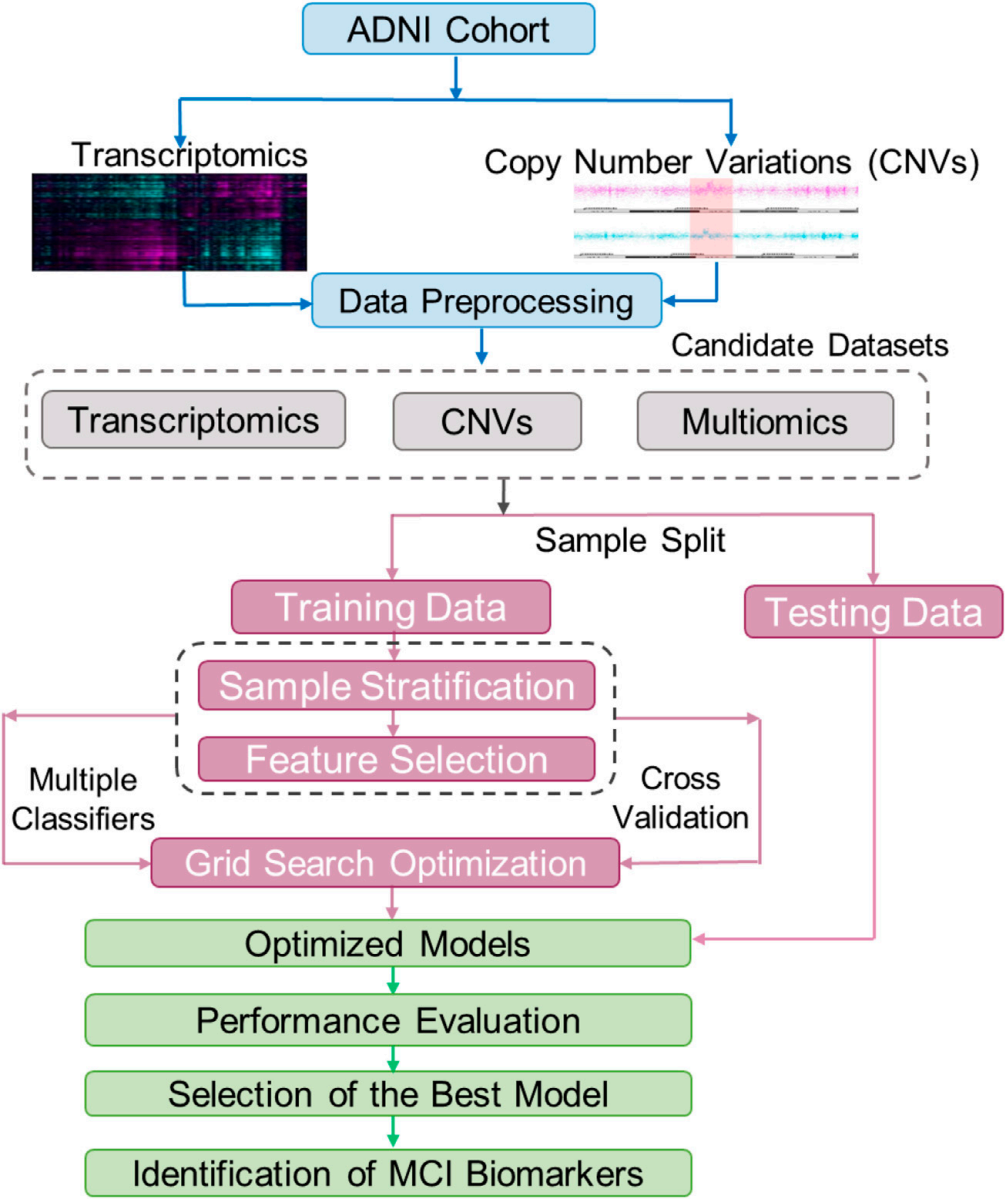
The workflow of building and testing models for classification of patients with Mild Cognitive Impairment (MCI). The raw transcriptomic and CNV data from the ADNI were normalized using a data processing pipeline. These normalized data were used to create three train/test datasets including one with multiomics (CNV and transcriptomics, also annotated as Exp + CNV), one with only CNV, and one with only transcriptomics (Exp). Each of these three datasets were split into training and testing datasets, and the training datasets were further split into training and validation. The training/validation sets were used for feature selection and hyperparameter optimization for 21 models (7 machine learning algorithms, each trained on the three different datasets). The testing datasets were used to evaluate the models. The best model was then used to find biomarkers for MCI.

We constructed three datasets to develop the prediction models using gene expression and CNV data from the ADNI cohort: 1) the transcriptomic (gene expression) data only, 2) the CNV data only and 3) the combination of the transcriptomic and CNV data (Figure 1). Since not all patients had both gene expression and CNV data from their blood samples, only the subset of 666 patients (245 NCI and 421 MCI) with both data types were retained to build the combination dataset (Table 1). Supplementary Figure S1 shows the frequencies of deletions, duplications, and multi‐allelic CNVs in the MCI and NCI groups.

**TABLE 1:** Demographic and clinical characteristics of ADNI participants in this study.

Dataset	Diagnosis	Number of patients	Gender			Race			Age	
			Femal (%)	Male (%)	P-value	Female (%)	Others (%)	P-value	Average age (SD)	P-value
Gene expression	NCI	258	51.9	48.1	9.30E-03	93.0	7.0	7.55E-01	74.4 (5.5)	5.49E-05
	MCI	437	41.6	58.4		93.6	6.4		72.3 (7.4)	
CNV	NCI	272	50.4	49.6	1.40E-02	93.0	7.0	7.57E-01	74.5 (5.6)	4.45E-05
	MCI	468	40.8	59.2		93.8	6.2		72.4 (7.5)	
Gene expression and CNV	NCI	245	51.0	49.0	1.22E-02	91.8	8.2	8.81E-01	74.5 (5.6)	8.30E-05
	MCI	421	40.9	59.1		92.4	7.6		72.3 (7.4)	

For each of the constructed datasets, we randomly allocated 90% of the samples for training and validation (10-fold cross validation) and the remaining 10% set aside for testing (Figure 1). In order to maintain balanced sample sizes in the NCI and MCI groups, during the tuning and training phases for the machine learning model, the NCI group was randomly over-sampled to match the number of MCI cases.

Before each time a model was trained, the training set for each omics dataset—gene expression and CNV (Exp + CNV)—was scaled between zero and one using the MinMaxScaler function from the scikit-learn python library. Afterwards, the testing sets were also scaled based on the min and max values of the associated training set. Gene expression and CNV data were then joined on patients to create the combination dataset for the classifiers we used (please see the following paragraph) except for MOGONET, which only takes the gene expression and/or CNV data without joining the input.

### Prediction of MCI patients

#### Binary classifiers

In order to capture both linear and non-linear data structures, we tested six classifiers, including three linear classifiers, Logistic Regression (LR), Logistic Regression with Stochastic Gradient Descent (LR-SGD) and Linear Support Vector Machine (LSVM); and three non-linear classifiers, Decision Tree (DT), Random Forest (RF) and eXtreme Gradient Boosting (XGBoost). In addition, we also assessed a recently developed multi-omics integrative method MOGONET (Multi-Omics Graph cOnvolutional NETworks), which uses a graphical convolutional neural network (GCN) approach and has been shown to outperform the other state-of-the-art supervised multi-omics integrative analysis approaches (Wang et al., 2021).

#### Feature selection

Since genome-wide biology data were used in this study, feature selection was performed on each data type in order to reduce overfitting. The genes and CNVs with variance less than 0.1 were removed from the feature set. We further removed one feature in each pair of the remaining features that were highly correlated (correlation coefficient >0.85) to reduce redundancy and multicollinearity. We then used scikit-learn’s Random Forest classifier and its built-in feature importance calculation, Gini importance, to assign each remaining feature an importance score. Next, Student’s t-test was used to calculate p-values of difference between MCI versu*s* NCI groups for each constructed dataset. Finally, the features with the p-values less than 0.05 and the feature importance score larger than 0 were selected to build the aforementioned models, except MOGONET. To build MOGONET models, the features were sorted by their student’s t-test p-values. The top *n* features with the smallest p-values among the features with importance score larger than 0 for each data type were retained for classification, where *n* was optimized for each data type through hyperparameter optimization.

#### Training, cross-validating and testing

Grid search has been used as a standard method for hyperparameter tuning in machine learning. It automates the process to exhaustively find the optimal combination of hyperparameters for a given machine learning model. In this study, we used this approach coupled with 10-fold cross validation to optimize the hyperparameters for each binary classifier. The proposed classification approach using grid optimization is illustrated in Figure 1. Supplementary Table S2 summarized the hyperparameters and their settings for the optimization. Since the area under the receiver operating characteristic curve score (ROC AUC score) is commonly used as the measure of the ability of a binary classifier to distinguish between classes, the 21 sets (each for a classifier trained on a given dataset) of hyperparameters that achieved the highest average AUC scores during the 10-fold cross validations were selected for training the models using the corresponding whole training-validation data. Then, the 21 optimized models were tested on the preserved testing data and their performances were evaluated using the standard prediction performance metrics including AUC, accuracy, precision, sensitivity and specificity. Among the 21 final models, the model with the best AUC on testing data was considered the best prediction model.

### Identification of important features for the prediction of MCI patients

We used the permutation feature importance, a model agnostic interpretability method, to identify important features for the prediction by measuring how much the model performance deteriorates when the values of a particular feature are randomly shuffled or permuted while keeping other variables unchanged. The features with permutation feature importance scores larger than 0 from the best prediction model were considered as the important features for further analysis.

### Analysis of the correlation between the predictive features and the clinical and neuropsychological traits

To assess the relevance of the identified important features to the clinical and neuropsychological traits of MCI patients, we performed Spearman’s correlation analysis. The association results were adjusted with false discovery rate (FDR) (Benjamini and Hochberg, 1995). The data for the clinical and neuropsychological variables were taken from 12 clinical and neuropsychological tests that are clinically helpful in diagnosing MCI patients which were performed on both MCI and NCI patients. These test include Clinical Dementia Rating Sum of Boxes (CDRSB), Alzheimer’s Disease Assessment Scale-Cognitive subscale 11 (ADAS11), Alzheimer’s Disease Assessment Scale-Cognitive subscale 13 (ADAS13), Alzheimer’s Disease Assessment Scale-Q4:delayed word recall (ADASQ4), Mini-Mental State Examination (MMSE), Rey Auditory Verbal Learning Test Immediate (RAVLT_immediate), Rey Auditory Verbal Learning Test Learning (RAVLT_learning), Rey Auditory Verbal Learning Test Forgetting (RAVLT_forgetting), ADNI modified Preclinical Alzheimer’s Cognitive Composite with Digit Symbol Substitution (mPACCdigit), ADNI modified Preclinical Alzheimer’s Cognitive Composite with Trials B (mPACCtrialsB), Functional Activities Questionnaires (FAQ) and Logical Memory – Delayed Recall (LDELTOTAL).

### Functional enrichment analysis

The Database for Annotation, Visualization and Integrated Discovery (DAVID) was used for the functional enrichment analysis of the 111 most important genes identified from the gene expression dataset. The 111 genes were used as input to DAVID while all human genome genes were provided as a background gene set. For each annotation term, DAVID calculates the frequency of the annotation term appearing in the input gene list compared to the expected frequency based on the background gene set, using a Fisher Exact test to determine enrichment significance (Huang et al., 2009).

## Results

### Cohort characteristics

The demographic and diagnostic characteristics for the subjects included in this study are summarized in Table 1. 695 subjects (258 CNs and 437 MCIs) have gene expression data from blood samples. 740 subjects (272 CNs and 468 MCIs) were included in CNV analysis. 666 subjects (245 CNs and 421 MCIs) have both expression and CNV data. Statistical analysis shows that there is no significant difference in race between MCI and NCI groups, but there are significant differences across diagnosis types in gender and age in all three datasets (Table 1). Therefore, both gene expression and CNV data were further processed to eliminate the biases introduced by race, age and gender difference (see the Materials and Methods section for detailed information; Supplementary Table S1).

### Evaluation of prediction models

Seven classifiers (XGBoost, RF, DT, LR, LR-SGD, SVM and MOGONET) and three datasets (gene expression, CNV and the combination of both) were used to build MCI prediction models. The hyperparameters for each model were tuned using grid search during 10-fold cross-validation (Figure 1; Supplementary Table S2). After tuning, we obtained 21 optimized models, whose refined hyperparameters are depicted in Supplementary Table S3.

**FIGURE 2:**
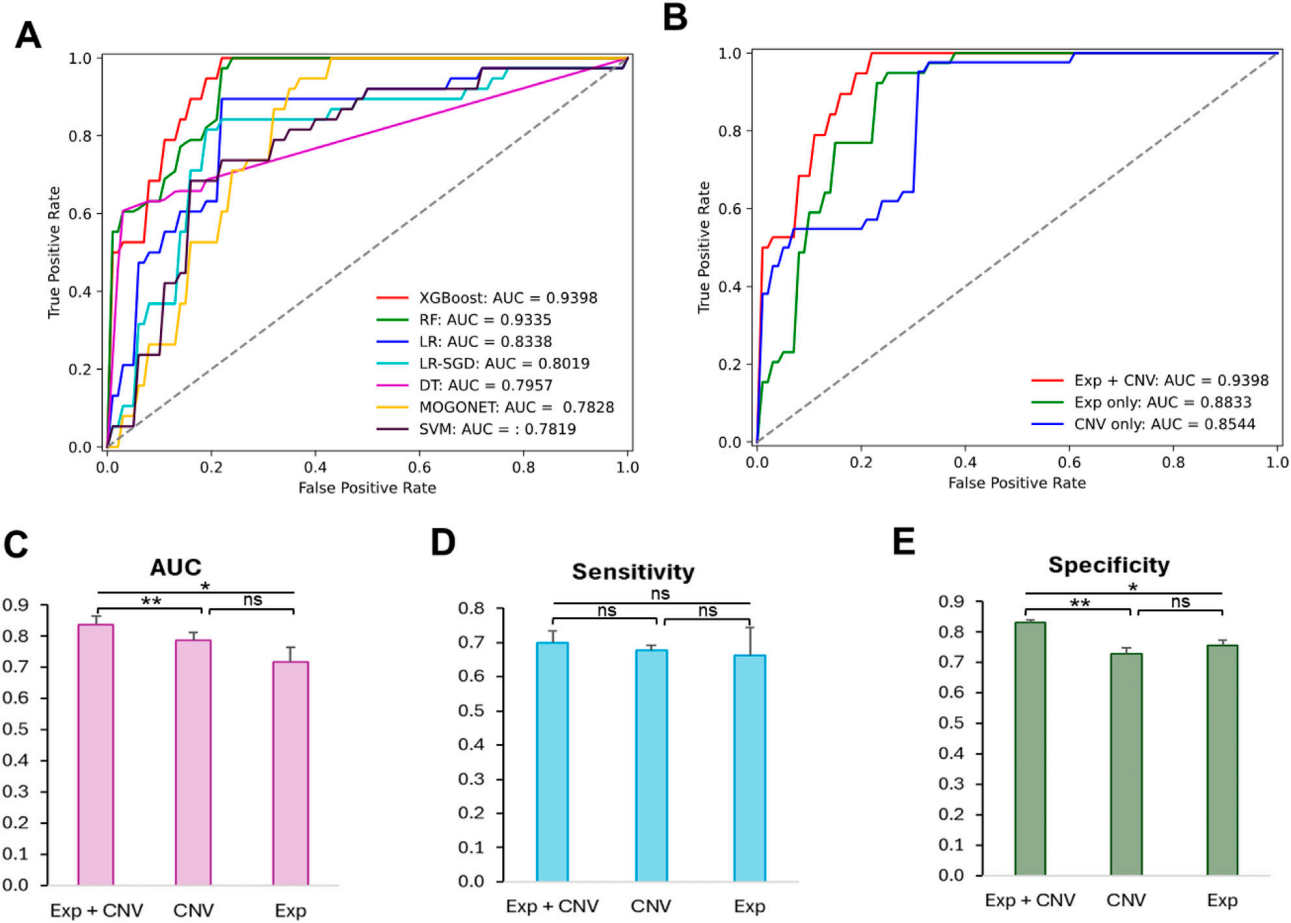
Comparison of the prediction performance of the 21 optimized models and the effectiveness of different datasets to predict MCI patients. **(A)** Receiver operating characteristic curves (ROC) of cross validation results from models trained on optimized hyperparameters found by grid search. **(B)** ROC curves of test results from XGBoost-based model trained on different datasets using optimized hyperparameters. **(C)** Comparison of AUC scores among classifiers trained on Exp + CNV, CNV and Exp. **(D)** Comparison of sensitivity scores among classifiers trained on Exp + CNV, CNV and Exp. **(E)** Comparison of specificity scores among classifiers trained on Exp + CNV, CNV and Exp. Paired two tailed student t test was used for the statistic test. p < 0.05 *, p < 0.01 **, ns, not significant; Exp, gene expression data; CNV, copy number variation; Exp + CNV, combination of both Exp and CNV data.

We then evaluated the performance of these models using the randomly preserved test data. Consistent with previously reported studies that the models using multi-omics data often perform better than using single-omics data (12, 14–17), the average AUC of the group of models trained on both expression and CNV data is significantly higher than the other two groups. No significant difference in AUC score was observed between models using gene expression data only or CNV data only (Figure 2C).

Among all 21 optimized models, the XGBoost based model using both expression and CNV data has the highest AUC score (0.9398) evaluated on the testing data (Table 2; Figures 2A,B). Moreover, this model also has the best performance measurements in accuracy (0.8289), precision (0.8788) and specificity (0.8947). Interestingly, RF trained on expression data only achieved the highest sensitivity (0.9744), which measures the model’s ability to designate an individual with MCI as positive, but suffered from low specificity (0.6750), which measures the model’s ability to designate an individual who does not have a disease as negative.

**TABLE 2:** Prediction performances of the 21 optimized models.

Dataset	Classifier	CV	Testing				
		AUC (SD)	AUC	Accuracy	Precision	Sensitivity	Specificity
Exp + CNV	XGBoost	0.8974 (0.0314)	**0.9398**	**0.8289**	**0.8788**	0.7632	**0.8947**
	RF	0.9029 (0.0267)	0.9335	0.8026	0.8485	0.7368	0.8684
	LR	0.7740 (0.0583)	0.8338	0.7368	0.7647	0.6842	0.7895
	LR-SGD	0.7589 (0.0626)	0.8019	0.8158	0.8000	0.8421	0.7895
	DT	0.7641 (0.0398)	0.7957	0.7632	0.8571	0.6316	**0.8947**
	MOGONET	0.7608 (0.0629)	0.7828	0.6816	0.7421	0.5579	0.8053
	SVM	0.7510 (0.0667)	0.7819	0.7368	0.7647	0.6842	0.7895
Exp only	XGBoost	0.8325 (0.0448)	0.8833	0.8101	0.7857	0.8462	0.7750
	RF	0.8301 (0.0291)	0.8154	0.8228	0.7451	**0.9744**	0.6750
	LR	0.6811 (0.0371)	0.6045	0.6329	0.6562	0.5385	0.7250
	LR-SGD	0.6823 (0.0413)	0.6083	0.6456	0.6774	0.5385	0.7500
	DT	0.7059 (0.0657)	0.8282	0.8228	0.8205	0.8205	0.8250
	MOGONET	0.7468 (0.0566)	0.6841	0.6051	0.6467	0.4410	0.7650
	SVM	0.6987 (0.0377)	0.6013	0.6329	0.6786	0.4872	0.7750
CNV only	XGBoost	0.9003 (0.0394)	0.8544	0.6824	0.6923	0.6429	0.7209
	RF	0.9202 (0.0327)	0.8882	0.7529	0.7561	0.7381	0.7674
	LR	0.7105 (0.0641)	0.7494	0.7412	0.7632	0.6905	0.7907
	LR-SGD	0.6791 (0.0599)	0.7259	0.6353	0.6279	0.6429	0.6279
	DT	0.7925 (0.0569)	0.7769	0.7412	0.7500	0.7143	0.7674
	MOGONET	0.7452 (0.0501)	0.7740	0.6847	0.6952	0.6429	0.7256
	SVM	0.6943 (0.0780)	0.7481	0.6824	0.6829	0.6667	0.6977

The bold values indicate the best values for each testing metric.

To understand whether this is a general trend that gene expression data may do better at improving prediction sensitivity, we examined the difference in the means of sensitivity among the groups of models trained on the gene expression data only, the CNV data only, or both the gene expression and CNV data. As shown in Figures 2C,D, the model based on the gene expression and CNV data has the best performance though there is no significant difference in prediction sensitivity among these groups.

We then further explored the prediction specificity difference among the models learning from CNVs, expression, and both data types. Figure 2E indicated that the models trained on both data types have significantly higher specificity than the models trained on only expression or CNV data, but there is no difference between the latter two groups of models.

Although only less than 9% of the samples were from non-Caucasian and all the features were corrected for age, sex and race to minimize the impact of those co-variates, some subtle population differences may not be completely removed. Therefore, we additionally investigated the impact of subtle population differences on the prediction performance. To do this, we trained and tested XGBoost, the best performing classifier for the complete dataset, based on the gene expression and CNV data from only the Caucasian subjects. We observed a slight improvement in predicting Caucasian MCI cases, with an AUC score of 0.9484, an accuracy of 0.8732, a precision of 0.9062, a sensitivity of 0.8266, and a specificity of 0.9167, suggesting that population structures have a small but noticeable impact on prediction performance.

Together, our results suggested that CNV data are as informative as gene expression for characterizing MCI patients, and the combination of both data types synergistically boosted the performance of the models.

### Identification and characterization of important features for the prediction of MCI patients

**FIGURE 3:**
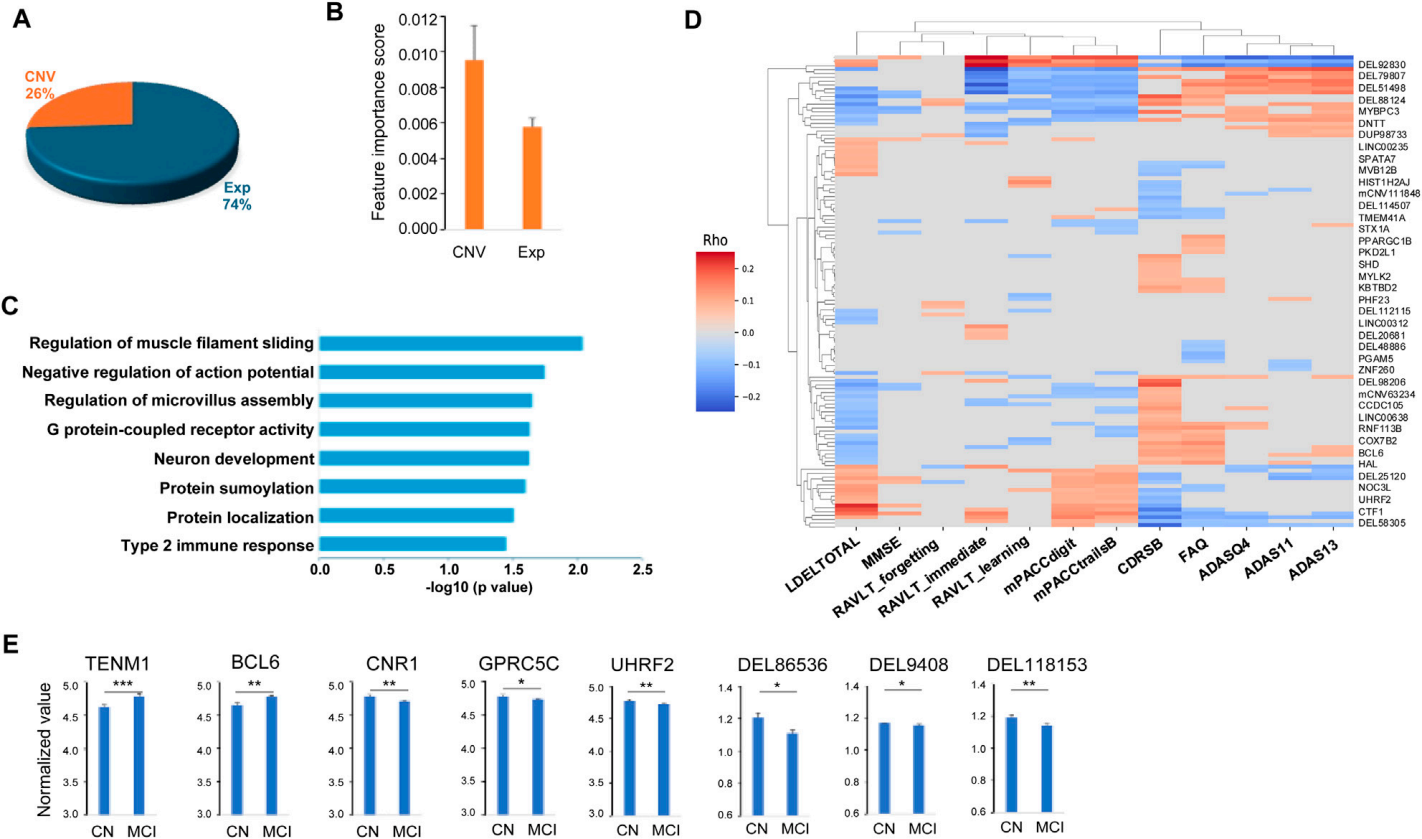
Characteristics of the 149 important features for predicting MCI. **(A)** Pie chart of the 149 important features including 111 genes (Exp) and 38 copy number variation (CNV). **(B)** Distributions of the important scores of the 111 genes and 38 CNVs. **(C)** Functional pathways enriched in the 111 genes important for predicting MCI. **(D)** Heatmap of the correlations between the selected important features and the clinical and neuropsychological traits. **(E)** Examples of the important features that can differentiate no cognitive impairment (NCI) and mild cognitive impairment (MCI) groups. Two tailed student t test was used for the statistical test. p < 0.05 *, p < 0.01 **, p < 0.001 ***.

As described in the Materials and Methods section, we obtained the importance scores from the best MCI patient prediction model. In total, there are 149 features with feature importance scores larger than 0 (i.e., positively contributing to MCI classification), and thus these features were considered as important features for MCI patient prediction. Among these important features, 111 (74%) are genes from the gene expression dataset and 38 are genomic locations (26%) from the CNV dataset (Figure 3A; Supplementary Table S4). While the majority of the important features are from the gene expression data, the average feature importance score of the CNV features is significantly (p = 1.68E-9) higher than the gene expression features (Figure 3B).

Functional enrichment analysis of the predicted genes was performed using the Database for Annotation, Visualization and Integrated Discovery (DAVID) (Huang et al., 2009). The one hundred and eleven important genes were enriched in a number of pathways including neuron development (including *TENM1*, *UGCG* and *CTF1*), cell communication and movement within and between neurons and other cell types, such as negative regulation of action potential (*SUMO1* and *CNR1*), regulation of microvillus assembly (PODXL, FSCN1), and regulation of muscle filament sliding (*MYBPC3*, *MYLK2*) (Figure 3C). The identified important genes are also enriched in the pathways that are important in development of cognitive impairment. For example, G protein-coupled receptor activity pathway, including important features (*TAS2R20*, *CNR1, OR4K2, KISS1R, FFAR3, OR2B11, OR13D1, OR6Q1* and *GPRC5C*), is essential in neurodegenerative diseases such as AD and Parkinson’s disease (Wong et al., 2023; Schöneberg et al., 2004; Guimaraes et al., 2021). Type 2 immune response pathway (*TRAF3IP2* and *BCL6*) plays an important role in AD pathology such as amyloid deposition (Marsh et al., 2016; Van Hoecke et al., 2024). Among the top-ranked biomarker genes, a number of them have been previously implicated in AD. For instance, *KISS1R* (KISS1 receptor) mediates the effects of kisspeptin, a polypeptide encoded by the KISS1 gene, with KISS1/KISS1R interactions in the brain suggested to exert a neuroprotective effect against AD (Panda et al., 2023). *EIF3H* has been identified as one of six novel biomarkers for AD through an integrated Weighted Gene Coexpression Network Analysis (WGCNA) (Zhang et al., 2021). PODXL (Podocalyxin) is a protein required in maintaining the blood-brain barrier (BBB) during acute inflammation (Cait et al., 2019). As BBB breakdown is thought to contribute to neurodegenerative diseases such as AD (Sweeney et al., 2018), PODXL may play a crucial role in AD pathogenesis, potentially through its impact on BBB integrity and neuroinflammation. *CCDC102B* has been suggestively associated with plasma amyloid levels through whole-exome sequence-based rare variant association tests in African and European Americans (Simino et al., 2017). Additionally, *TET1*, a member of the TET family of enzymes that dynamically regulate epigenetic modifications in response to environmental conditions, has been implicated in AD pathogenesis. Its loss may exacerbate AD-related pathologies, impacting gene expression and cognitive function (Armstrong et al., 2023).

After mapping the important CNV genomic locations to their affected genes, we found that these genes were often associated with neurodegenerative diseases such as AD. For example, DEL118153 is a deletion variant of *NTSR1* (neurotensin receptor 1) gene. NTSR1 belongs to the large superfamily of G-protein coupled receptors. The activation of *NTSR1* facilitates neuronal excitability and spatial learning and memory (Xiao et al., 2014), and the polymorphism in NTSR1 is associated with the impairment of the working memory function, which is usually affected early during the course of AD (Li et al., 2011; Jahn, 2013). DEL94608 is a *VTI1B* (vesicle transport through interaction with t-SNAREs 1B) deletion variant. It has been reported that lack of *VTI1B* led to significant impairments in neuronal development and synaptic transmission. *VTI1B* is also involved in AD pathogenesis such as Aβ plaque and Tau aggregation and accumulation (Kunwar et al., 2011; Bollmann et al., 2022; Tang et al., 2022; Emperador-Melero et al., 2019; Margiotta, 2021). DEL48886 includes a *NCOA7* (nuclear receptor coactivator 7) gene region deletion. *NCOA7* is an important V-ATPase regulatory protein in the brain. Neurons lacking *NCOA7* exhibit altered development and NCOA7 deletion animals exhibited abnormal neuronal patterning defects (Castroflorio et al., 2021). DEL86536 affects *MGAT4C* (MGAT4 family member C) gene. Deletion of MGAT4C has been associated with neurocognitive disorders (Bliskunova et al., 2021).

To understand how our model distinguishes MCIs from CNs, firstly, we compared the values of the identified 149 important features between MCI and NCI groups, and found that the majority (54.4%) including 60.5% of the CNVs and 52.3% of the gene features are discriminative (p < 0.05), as illustrated in Figure 3E. Secondly, we characterized the association of the identified important features with the clinical and neuropsychological traits used for the diagnosis of MCI, and observed 81.2% of the features including 94.7% of the CNVs and 76.6% of the gene features had statistically significant correlation (FDR <0.05, Spearman’s correlation) with at least one of the 12 traits (CDRSB, ADAS11, ADAS13, ADASQ4, MMSE, RAVLT_immediate, RAVLT_learning, RAVLT_forgetting, mPACCdigit, mPACCtrialsB, FAQ and LDELTOTAL) (Figure 3D). Together, our results suggest that our prediction model was able to shed light on the molecular characteristics of MCI, and the important features that are associated with the clinical traits of MCI patients.

## Discussion

In this study, we built an XGBoost based prediction model using blood multi-omics data with good prediction performance (AUC = 0.9398). To our knowledge, this is the first MCI/AD prediction model utilizing CNV data derived from patient blood samples. We also identified a panel of 149 CNV and gene expression features as potential biomarkers based on their importance in MCI classification. These features are involved in neuron development, movement and communication, and most of them are significantly correlated to clinical and neuropsychological traits.

Consistent with previous reports, our study demonstrated that models using multi-omics data (CNV and gene expression) performed better than models trained on single-omics data. Through analysis of the performance matrix, we revealed that the reason behind the performance boost could be due to the improvement of the model specificity by the synergistic effect of different data types. Literature search revealed that our XGBoost-based model outperformed all reported AD/MCI prediction models utilizing genomic data from patient blood samples, such as the models created by Oriol et al., AlMansoori et al., Lee and Lee, and Venugopalan et al. with AUC or accuracy scores within 0.62–0.72 range (Table 3).

**TABLE 3:** Performance comparisons with published methods.

Method	Classification type	Classification group	Dataset	AUC	Accuracy	References
XGBoost	Binary	MCI vs. NCI	CNVs and blood gene expression	0.9398	0.8289	This study
SVM	Binary	MCI vs. NCI	28 blood plasma proteomic biomarkers	0.91	N/A	O'Bryant et al. (2022)
B-HEALED	Binary	AD vs. NCI	81 multiomics blood-based biomarkers	0.819	N/A	Souchet et al. (2024)
BSWiMS-LASSO-RPART ensemble	Binary	AD vs. NCI	SNPs	0.719	0.677	De Velasco Oriol et al. (2019)
AdaBoost	Binary	MCI/AD vs. NCI	SNPs and clinical data without clinical scores	0.63	0.67	AlMansoori et al. (2024)
Random Forest	Binary	MCI/AD vs. NCI	Blood gene expression and clinical data without clinical scores	0.65	0.65	AlMansoori et al. (2024)
SVM	Binary	AD vs. NCI	Blood gene expression	0.62	N/A	Lee and Lee (2020)
Deep learning models	Binary	MCI/AD vs. NCI	SNPs	N/A	0.66	Venugopalan et al. (2021)

Through analysis of the performance matrix, we revealed that the reason behind the performance boost by multi-omics data compared to single-omic data could be due to the improvement of the model specificity by the synergistic effect of different data types. Our literature search results revealed that our XGBoost-based model also outperformed all reported AD/MCI prediction models utilizing data from patient blood samples. Table 3 shows that the studies using blood genomics data from ADNI (Oriol et al., AlMansoori et al., Lee and Lee, and Venugopalan et al.) and studies using plasma biomarkers (Souchet et al. and O’Bryant et al., AUC = 0.819 and 0.91, respectively) all reported lower AD/MCI prediction performances than our model.

We were able to use genomic data to achieve state-of-the-art MCI prediction in large part due to our ability to denoise the data, which was done using our internally developed data preprocessing pipeline. In addition, our study found CNV data could be as informative as gene expression data to classify MCI patients from normal controls. Indeed, the models built on only CNV data achieved good performances with AUC scores 0.8544 and 0.8882 for XGBoost and RF- based models, respectively. Therefore, data at a genome structure level such as CNVs, which have been overlooked in previous MCI prediction studies, can provide valuable information to characterize MCI patients, and including CNV data in our dataset likely contributed to an overall superior performance.

In this study, we used a large number of MCI patient samples (437 gene expression and 468 CNV) to build the prediction model, but the size of normal control samples is substantially smaller (258 and 272 for gene expression and CNV, respectively) (Table 1). Although we used an up-sampling strategy to address the class imbalance in our data, our model might be at the risk of overfitting, if the current normal control data do not adequately capture the reality. Therefore, it will be helpful to test our model using additional blood sample-derived data to confirm findings, which could open the way to develop a better classification algorithm for the prediction of MCI patients.

## Data Availability

Publicly available datasets were analyzed in this study. This data can be found here: ADNI.
