# Supplementary material for: Prediction of mild cognitive impairment using blood multi-omics data
Source: Front Genet. 2025 May 26;16:1552063. doi: 10.3389/fgene.2025.1552063 (PMC12146786; doi:10.3389/fgene.2025.1552063)
Supplement: Supplementary file 1 [file Supplementaryfile1.pdf]

Supplementary Tables

Supplementary Table S1. The original sample sizes for gene expression and copy number variation datasets.

Dataset	NCI	AD	MCI		Total
			EMCI	LMCI	
Gene expression	258	40	212	225	735
Copy number variation	272	45	230	238	785

Supplementary Table S2. Hyperparameter configuration of the classifiers.

Classifier	Hyperparameter
XGBOOST	learning_rat: [0.01, 0.1, 0.3, 0.5]
	max_depth: [2, 5, 10, 15]
	subsample: [0.5, 0.8, 1]
RF	criterion: ['entropy', 'gini']
	min_samples_split: [2, 5, 10]
	min_samples_leaf: [1, 2, 3, 4, 5]
	bootstrap: [True, False]
DT	criterion: ['entropy', 'gini']
	min_samples_split: [2, 5, 10]
	min_samples_leaf: [1, 2, 3, 4, 5]
LR	C: [0.01, 0.1, 1, 10, 100, 1000, 10000]
	penalty: ['l1', 'l2']
	solver: ['liblinear']
LR-SGD	loss: ['log_loss']
	penalty: ['l1', 'l2', 'elasticnet']
	alpha: [0.00001, 0.0001, 0.001, 0.01, 0.1, 1, 10]
SVM	C: [0.01, 0.1, 1, 10, 100, 1000, 10000]
	kernel: ['linear']
MOGONET	adj_parameters: [2, 5, 10]
	num_epoch_pretrain: [100]
	num_epoch: [3000, 6000]
	lr_e_pretrain: [1e-5]
	lr_e: [5e-5, 1e-3]
	lr_c: [5e-5, 1e-3]
	num_exp_feature: [500, 700]
	num_cnv_feature: [300, 500]

Supplementary Table S3. Optimized hyperparameters.

Classifier	Exp + CNV	Exp	CNV
**XGBoost**	[100, 0.1, 5, 0.8]	[100, 0.01, 10, 1]	[100, 0.1, 15, 1]
**RF**	[100, 'sqrt', 'entropy', 2, 1, False]	[100, 'sqrt', 'entropy', 10, 3, False]	[100, 'log2', 'entropy', 2, 2, False]
**LR**	[10000, 'l1', 'liblinear']	[1, 'l2', 'liblinear']	[10000, 'l1', 'liblinear']
**LR-SGD**	['log_loss', 'l2', 0.001]	['log_loss', 'elasticnet', 0.0001]	['log_loss', 'l2', 0.01]
**DT**	['entropy', 2, 4]	['gini', 10, 2]	['gini', 10, 4]
**MOGONET**	[2, 600, 200, 6000, 0.001, 500, 300]	[2, 600, 200, 6000, 5e-05, 500, 300]	[2, 400, 200, 6000, 0.001, 700, 300]
**SVM**	[0.1, 'linear']	[10, 'linear']	[0.1, 'linear']

Supplementary Table S4. Important features for the classification of MCI patients.

Gene	Data type	CNV affected gene	Importance score	pval (MCI vs. NCI)
mCNV63234	CNV		7.24E-02	1.18E-03
DEL25120	CNV	ALG1L2,FAM86HP	3.29E-02	1.17E-01
KISS1R	Exp		2.68E-02	2.49E-02
LINC00638	Exp		2.65E-02	9.48E-03
CPB1	Exp		2.36E-02	7.62E-02
mCNV37875	CNV		1.76E-02	6.76E-03
EIF3H	Exp		1.72E-02	2.51E-02
SFTA2	Exp		1.62E-02	6.15E-03
DEL58305	CNV		1.59E-02	2.22E-03
PPP1R2P1	Exp		1.55E-02	6.79E-03
DEL79807	CNV	FADS2	1.49E-02	1.76E-01
DEL10053	CNV	SNTG2	1.35E-02	3.78E-01
DEL75157	CNV	MINPP1	1.34E-02	1.35E-01
PODXL	Exp		1.34E-02	1.30E-03
CCDC102B	Exp		1.19E-02	2.22E-01
OR2B11	Exp		1.17E-02	2.63E-02
TET1	Exp		1.16E-02	1.16E-01
MYLK2	Exp		1.13E-02	2.69E-02
MYBPC3	Exp		1.11E-02	3.74E-03
DSCC1	Exp		1.10E-02	2.57E-02
SMCO2	Exp		1.09E-02	3.04E-02
DEL98206	CNV		1.07E-02	8.00E-02
mCNV111848	CNV		1.05E-02	1.40E-01
COX7B2	Exp		1.04E-02	1.99E-02
DEL114507	CNV	ZFP14	1.03E-02	4.29E-02
COQ9	Exp		9.86E-03	9.75E-02
FAM92B	Exp		9.74E-03	1.63E-01
DEL112115	CNV		9.73E-03	9.40E-03
NUF2	Exp		9.62E-03	3.57E-02
P2RY12	Exp		9.60E-03	3.93E-02
CYP4A22	Exp		9.60E-03	7.93E-02
IRF1	Exp		9.40E-03	1.51E-01
PPARGC1B	Exp		9.11E-03	5.83E-02
mCNV99997	CNV		8.99E-03	1.45E-02
STX1A	Exp		8.99E-03	6.82E-02
DEL48886	CNV	NCOA7	8.68E-03	3.78E-03
TENM1	Exp		8.36E-03	3.60E-04
RAB28	Exp		8.32E-03	1.14E-02
DEL86536	CNV	MGAT4C	8.28E-03	1.43E-02
ACMSD	Exp		8.11E-03	3.80E-03
ZNF835	Exp		8.06E-03	2.30E-02
DEL18487	CNV		7.92E-03	2.06E-02
TNFRSF18	Exp		7.90E-03	3.83E-03
PAGE1	Exp		7.73E-03	8.25E-02
ZBTB9	Exp		7.47E-03	8.01E-02
DEL14568	CNV	RGPD3	7.46E-03	5.57E-02
RAVER2	Exp		7.28E-03	2.75E-02
ASB16-AS1	Exp		7.15E-03	1.30E-02
OR4K2	Exp		7.07E-03	2.37E-03
CCDC105	Exp		6.97E-03	2.71E-02
DEL94608	CNV	VTI1B	6.90E-03	1.82E-02
DEL57047	CNV	CNOT4	6.90E-03	3.66E-02
FAM76A	Exp		6.81E-03	2.79E-02
DEL88124	CNV		6.78E-03	3.05E-03
TMEM41A	Exp		6.75E-03	1.33E-03
HSD3B1	Exp		6.58E-03	1.18E-01
DEL64469	CNV	ZNF7	6.45E-03	1.87E-01
FAM47C	Exp		6.40E-03	3.67E-01
DEL115590	CNV	ZNF701	6.38E-03	3.91E-02
HIST1H2AJ	Exp		6.24E-03	4.33E-02
SUMO1	Exp		5.96E-03	7.61E-02
DEL27429	CNV	CPN2	5.82E-03	1.44E-01
ADRB3	Exp		5.82E-03	5.93E-02
DEL116171	CNV		5.77E-03	5.07E-02
GSTA3	Exp		5.76E-03	1.33E-01
CTF1	Exp		5.75E-03	8.08E-02
DNTT	Exp		5.74E-03	1.26E-01
DEL51498	CNV	ISPD(CRPPA)	5.56E-03	1.20E-02
HAL	Exp		5.54E-03	2.93E-02
TRAF3IP2	Exp		5.49E-03	7.99E-02
NOC3L	Exp		5.44E-03	1.81E-02
KIFC3	Exp		5.43E-03	1.86E-01
NDUFC1	Exp		5.29E-03	4.01E-02
TAS2R20	Exp		5.29E-03	2.44E-02
SCO1	Exp		5.09E-03	2.55E-01
DEL92830	CNV		5.08E-03	1.89E-02
PGAM5	Exp		5.05E-03	2.12E-01
DEL115967	CNV	PTPRH	5.03E-03	5.50E-02
KRT222	Exp		4.96E-03	3.72E-02
DEL20643	CNV	SSUH2	4.93E-03	3.61E-02
LSMEM2	Exp		4.85E-03	2.17E-02
FGF3	Exp		4.84E-03	7.73E-02
FSCN1	Exp		4.67E-03	8.23E-02
SCN9A	Exp		4.65E-03	2.23E-01
KRTAP4-9	Exp		4.56E-03	8.08E-02
ARHGAP35	Exp		4.51E-03	2.89E-02
mCNV116412	CNV		4.50E-03	5.03E-02
KRTAP10-10	Exp		4.49E-03	2.95E-02
BCL6	Exp		4.44E-03	5.61E-03
FERD3L	Exp		4.38E-03	4.34E-02
DEL4824	CNV	VANGL1	4.29E-03	2.15E-01
UHRF1BP1	Exp		4.21E-03	7.01E-02
PKD2L1	Exp		4.18E-03	2.81E-01
mCNV43702	CNV		4.18E-03	3.44E-02
DEL1122	CNV	CDA	4.03E-03	3.43E-02
UHRF2	Exp		4.01E-03	2.97E-03
TMEM242	Exp		3.96E-03	6.69E-02
CETN2	Exp		3.84E-03	9.04E-02
DBX2	Exp		3.84E-03	2.11E-01
RNF113B	Exp		3.76E-03	3.73E-02
DEL63732	CNV	WDYHV1	3.57E-03	5.52E-02
SMARCD3	Exp		3.46E-03	4.30E-02
CRISPLD2	Exp		3.31E-03	3.40E-03
DEL64660	CNV		3.31E-03	1.49E-02
DEL106133	CNV		3.28E-03	1.21E-02
UGCG	Exp		3.10E-03	2.95E-01
DEL118153	CNV	NTSR1	2.85E-03	2.44E-02
MVB12B	Exp		2.83E-03	4.67E-02
AMZ1	Exp		2.71E-03	7.47E-02
NSF	Exp		2.71E-03	5.33E-02
PGLS	Exp		2.71E-03	1.61E-01
LUM	Exp		2.69E-03	1.29E-01
LRG1	Exp		2.69E-03	8.56E-02
NMT2	Exp		2.68E-03	9.53E-02
CAVIN1	Exp		2.68E-03	3.04E-02
CLDN1	Exp		2.51E-03	1.22E-02
KBTBD2	Exp		2.48E-03	1.02E-02
LINC00235	Exp		2.47E-03	4.09E-02
FAM25A	Exp		2.33E-03	1.85E-02
PRM2	Exp		2.32E-03	5.11E-02
ERICH2	Exp		2.29E-03	3.81E-01
CDH20	Exp		2.18E-03	1.95E-01
KLHL40	Exp		2.07E-03	3.66E-01
HOXD1	Exp		2.05E-03	3.14E-02
DEL20681	CNV		1.99E-03	5.08E-03
GATS	Exp		1.90E-03	5.63E-03
BCORL1	Exp		1.89E-03	2.44E-02
PHF23	Exp		1.86E-03	3.95E-03
RIOK1	Exp		1.81E-03	1.44E-01
FFAR3	Exp		1.74E-03	1.21E-01
ERCC4	Exp		1.70E-03	2.18E-01
EXT1	Exp		1.55E-03	2.63E-02
SPAG17	Exp		1.31E-03	1.00E-02
GPRC5C	Exp		1.20E-03	1.24E-02
MCM10	Exp		1.05E-03	5.03E-02
SPATA7	Exp		1.03E-03	2.60E-02
PARP4	Exp		1.01E-03	7.06E-02
DEL100266	CNV	HBZ,HBM	8.99E-04	2.01E-02
DUP98733	CNV	ALDH1A2,LIPC	8.58E-04	5.38E-02
ZNF260	Exp		6.26E-04	1.63E-01
SHD	Exp		5.78E-04	1.14E-02
ROM1	Exp		4.89E-04	1.31E-01
FBXO31	Exp		1.75E-04	1.15E-01
CLPS	Exp		1.26E-04	6.84E-02
CNR1	Exp		9.47E-05	5.98E-03
SCAND2P	Exp		3.03E-05	7.65E-02
LINC00312	Exp		2.76E-05	1.84E-02
OR6Q1	Exp		1.71E-05	4.90E-02
OR13D1	Exp		7.89E-06	9.79E-02

Supplementary Figure

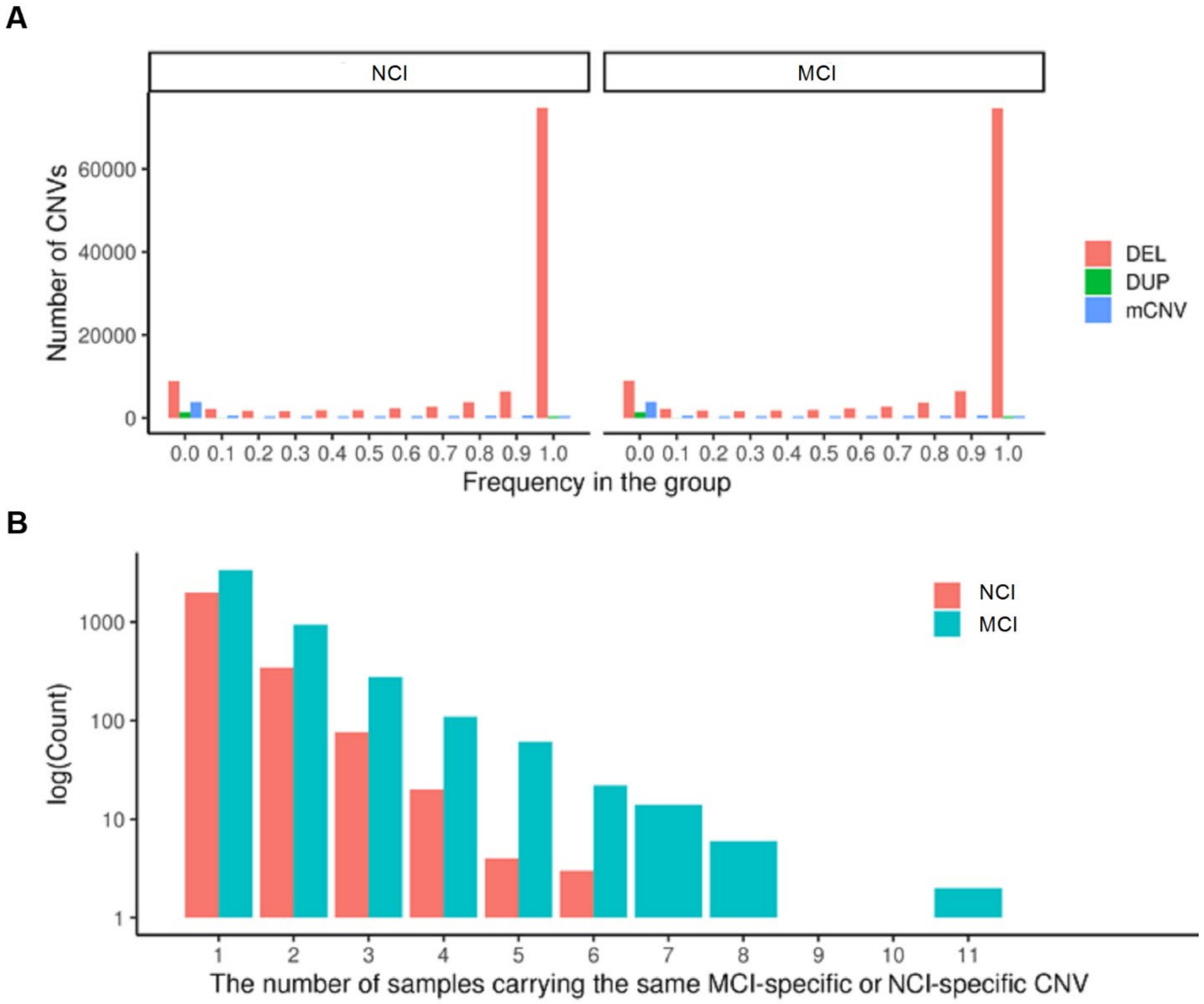

Supplemental Figure S1. CNV site frequency in the ADNI cohort. A. CNV frequencies of deletions, duplications, and multi-allelic CNVs across the MCI and NCI (control) groups. The frequency is calculated in each group separately. **B.** Site frequencies of MCI-specific and NCI-specific CNVs. When the frequency of a CNV in the MCI group is greater than 0 and its frequency in the NCI group is zero, the CNV is classified as MCI-specific; conversely, when the frequency of a CNV in the NCI group is greater than 0 and its frequency in the MCI group is zero, the CNV is classified as NCI-specific**.**
